# Microwave Radiometers for Fire Detection in Trains: Theory and Feasibility Study †

**DOI:** 10.3390/s16060906

**Published:** 2016-06-17

**Authors:** Federico Alimenti, Luca Roselli, Stefania Bonafoni

**Affiliations:** Department of Engineering, University of Perugia, via G. Duranti 93, 06125 Perugia, Italy; luca.roselli@unipg.it (L.R.); stefania.bonafoni@unipg.it (S.B.)

**Keywords:** automatic fire detection, microwave sensors, microwave radiometers, remote sensing, moving vehicles, microwave electronics

## Abstract

This paper introduces the theory of fire detection in moving vehicles by microwave radiometers. The system analysis is discussed and a feasibility study is illustrated on the basis of two implementation hypotheses. The basic idea is to have a fixed radiometer and to look inside the glass windows of the wagon when it passes in front of the instrument antenna. The proposed sensor uses a three-pixel multi-beam configuration that allows an image to be formed by the movement of the train itself. Each pixel is constituted by a direct amplification microwave receiver operating at 31.4 GHz. At this frequency, the antenna can be a 34 cm offset parabolic dish, whereas a 1 K brightness temperature resolution is achievable with an overall system noise figure of 6 dB, an observation bandwidth of 2 GHz and an integration time of 1 ms. The effect of the detector noise is also investigated and several implementation hypotheses are discussed. The presented study is important since it could be applied to the automatic fire alarm in trains and moving vehicles with dielectric wall/windows.

## 1. Introduction

The precise localization of fire spots is an operational aspect of great relevance to cope with fires. A very promising approach is given by microwave radiometry [1,2,3,4]. Although lower performing in terms of spatial resolution than Infrared (IR) sensors, microwaves can penetrate layers of concrete material, vegetation and dense smoke, detecting a target even if it is invisible to an external observer [5,6,7]. This principle has also been exploited by several mm-wave imaging systems [8,9,10,11].

Nonetheless, in order to accurately model the microwave fire detection and assess the properties (emissivity, reflectivity and transmissivity) of obstacle materials, typically encountered in operational conditions, much work seems to be still be needed.

The purpose of this research is to perform a feasibility study about the microwave fire detection in moving trains. A theoretical model of the scene sensed by a microwave radiometer has already been carried-out in a previous paper [12]. This model accounts for the presence of both fire spots and wall-like obstacles, while exploiting a rigorous definition of the filling factor.

From the study in [12] emerges the possibility of detecting fires in vehicles by using microwave sensors. The main assumption is that part of the vehicle body is made of non metallic materials or that the vehicle has dielectric walls or windows. Metals, indeed, shield the electromagnetic radiation not allowing the signal to pass through. In addition, the emissivity of a metal surface is very low and, as a consequence, the microwave emissions of the heated walls is very low too.

In spite of the previous observation apparently limiting microwave fire detection in trains, there are lots of cases in which this technique can be successfully applied. First of all, there are a number of good wagons that are open or have a dielectric body (plastic or fiber-glass). Secondly, passenger trains have windows made of glass, the latter being quite a good dielectric from the point of view of microwave radiation. At this point, it is important to note that glass is not transparent at IR wavelengths, thus it is nearly impossible for an IR radiometer to look inside the train through a glass window (see also: http://www.youtube.com/watch?v=wsjjdwLFNIM). The fire in passenger compartments belongs to the enclosed fire cases. These fires present more problems with the detection because they usually develop slowly with limited flames, heat and smoke in the initial minutes after the ignition. As a consequence, early detection methods are particularly important for enclosed fires.

In this paper, for the first time, the theory of microwave radiometers devoted to the fire detection in moving vehicles, such as trains, is introduced. For passenger wagons, the basic idea is to have a fixed radiometer looking trough the wagon windows under a suitable angle. The following parameters are to be considered: train speed, observation distance and angle, observation frequency, antenna beam-width, and radiometer integration time.

The paper is organized as follows: after a system overview, the specifications of the radiometer will be defined. Then, a first-guess design of the antenna is provided and its equivalent noise temperature is estimated. With these data, the instrument resolution is computed. Finally, a system design, two implementation hypotheses are discussed, and the conclusions are drawn.

## 2. System Overview and Specifications

The working hypothesis of the present study is the adoption of a microwave/millimeter-wave radiometer for the remote sensing of fire in a moving train. A passenger train scenario is considered since it imposes the most severe constraints to the design of the radiometer hardware. According to [12], indeed, the radiometer is fixed and placed under a gate at a certain distance from the train. The instrument is able to scan the walls of each wagon, thanks to the train movement that happens at a known speed $v_{T}$. To observe the wagon interior, the radiometer will exploit the wagon windows that, being made of glass, are quite transparent to the microwave radiation. As a consequence of this detection approach, the radiometer has only a few dozen of milliseconds to perform the detection, and this imposes state-of-the-art performance on the hardware. For example, if the train speed is 300 km/h, a 1.2 m window is scanned in 14.4 ms by the radiometer antenna. This means that the instrument should be designed to provide a reliable fire detection in such a small amount of time.

A very important relationship exists between the train speed, the width of the wagon windows, the antenna beam width and the radiometer integration time. The latter is a very important instrument parameter similar to the exposition time of a common optical photo-camera. A quantitative description of this relationship can be found in [12]. From the qualitative point of view, however, it can be easily deduced that an increase of the train speed causes a reduction of the radiometer integration time and, as a consequence, a worse performance in terms of temperature resolution (this is discussed in Section 5). The main specifications of the microwave radiometer have been discussed in [12] and are summarized here in Table 1.

In this table, $f_{0}$ is the observation frequency, *B* is the equivalent noise bandwidth of the radiometer, $N_{b}$ is the number of antenna beams to be considered, $\Theta_{h}$ is the 3 dB beam-width of the antenna, *d* is the instrument to train distance, $v_{T}$ is the train speed, *τ* is the integration time, ΔT is the radiometric temperature resolution and $T_{A}$ indicated the range of antenna noise temperatures that can be measured by the sensor.

**Figure 1:**
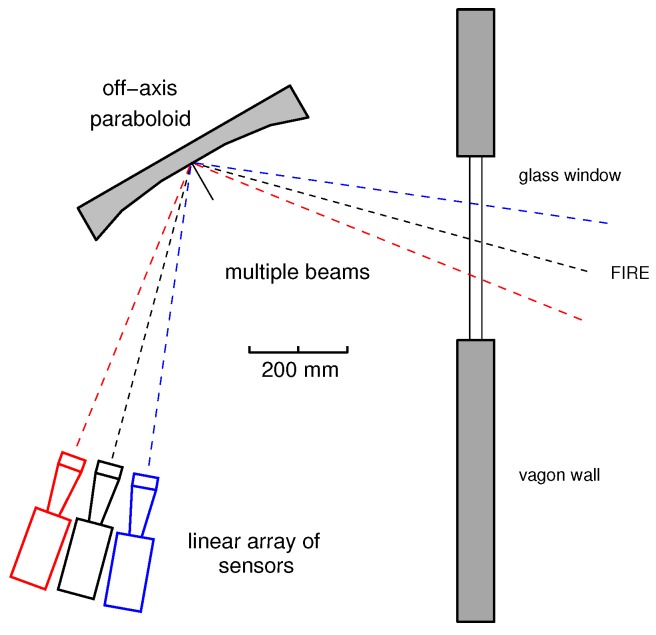
Concept of a multi-beam receiver with $N_{b}=3$. In this case, three independent radiometers are used for each beam. These beams are similar to the pixels of a common optical photo-camera. The secondary antennas (circular feed-horns in the figure) are mounted with a small angular offset in such a way as to produce the multiple beams. Only one main parabolic reflector is used here to the microwave radiation into each receiver.

The concept of a multi-beam receiver is illustrated in Figure 1, for the case of $N_{b}=3$. With such a technique, the scanned wagon window is divided in $N_{b}$ stripes and, as a consequence, an image can be formed with the vehicle movement. It is worth noticing here that the $N_{b}$ radiometers work in parallel, thus not affecting the time needed for the image formation. These radiometers are similar to the individual photo-diodes (one for each image pixel) equipping the sensor of a common optical photo-camera.

**Figure 2:**
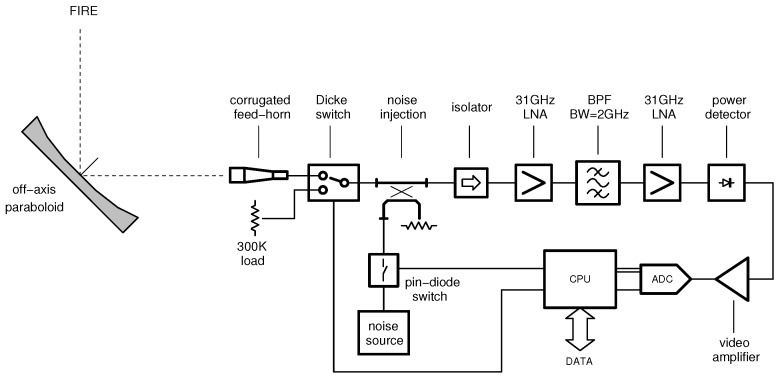
Schematic of the millimeter-wave radiometer module: a direct amplification architecture is assumed. The receiver gain is calibrated by noise injection, whereas the radiometer offset is calibrated using a reference load, working as a black-body, and a Dicke switch. Acronyms: Low Noise Amplifier (LNA); Band-Pass Filter (BPF); Bandwidth (BW); Analog-to-Digital Converter (ADC); Central Processing Unit (CPU).

A possible radiometer block diagram is reported in Figure 2. A direct amplification architecture is assumed because of its superior performance in terms of parametric stability with respect to heterodyne receivers [13]. The receiver gain is calibrated by noise injection, whereas the radiometer offset is periodically corrected using a reference load working as a black-body and an input switch (also referred to as Dicke switch). The noise source is typically implemented by means of an avalanche noise diode that, recently, was demonstrated in commercial Complementary Metal-Oxide Semiconductors (CMOS) technology [14]. Using such an approach, the whole calibration circuit can be miniaturized in a single chip (CMOS switches are well-known).

## 3. Antenna Design

At millimeter wavelengths, geometrical optics cannot be applied. One of the main assumptions of geometrical optics, indeed, is that the size of optical components is larger than the wavelength. At these frequencies, however, propagating waves can have the electric field with a Gaussian distribution. The optics in this domain are therefore called Gaussian optics (or quasi-optics), and these principles can be used for the sizing of the antenna.

The antenna design is handled in two steps. First, the Gaussian optics [15] is used to obtain an initial guess. Then, the design is refined exploiting an antenna design CAD tool, *i.e.*, GRASP 9 Student Edition (TICRA, Copenhagen, Denmark) [16,17].

The Gaussian modes are obtained as the solution of the the Huygens–Fresnel diffraction integral for the E-field distribution in free space. These modes are typically excited by a Gaussian source of radiation—for instance, a horn antenna. The main properties of Gaussian modes are the following. The propagation occurs along an axis, say the *z*-axis, with a plane phase front at z=0. The E-field maximum will be on the axis itself. Then, the amplitude will decay according to a Gaussian distribution. The distance from the *z*-axis to the points where the E-field falls to 1/e of the maximum will be defined as the beam size or beam radius. The minimum beam size is called the beam waist $w_{0}$ and is localized where the phase front is planar, *i.e.*, at z=0 with the above assumptions. At a certain frequency, the beam size *w* depends on two parameters only, namely the distance from the beam waist *z* and the beam waist radius:

(1) $$w\;\left(z\right)=w_{0}\;\sqrt{1+{\left(\frac{z}{z_{c}}\right)}^{2}}$$

where $z_{c}$ is the confocal distance. This parameter is, in practice, the distance where the antenna radiation can be approximated by the far-field conditions. The confocal distance is a function of both $w_{0}$ and the wavelength *λ*:

(2) $$z_{c}=\frac{\pi \;w_{0}^{2}}{\lambda }$$

The half power beam width $\Theta_{h}$ of the antenna is related to the far field divergence angle $\theta_{0}$ of the beam:

(3) $$\Theta_{h}=1.18\;\theta_{0}$$

which can be written in terms of both frequency and beam waist radius:

(4) $$\theta_{0}=\frac{\lambda }{\pi \;w_{0}}$$

As a result, the fundamental Gaussian mode parameters $w_{0}$ and $z_{c}$ can be found from the knowledge of $\Theta_{h}$. In the case of the antenna specifications stated in the previous paragraph (see Table 1), the Gaussian beam parameters are summarized in Table 2. The table has been extended to other frequencies of potential interest.

The first hypothesis for the antenna implementation is based on the usage of metallic reflectors. This is because of their well-proven robustness and mechanical strength as well as their high optical performance. The case of a single parabolic dish fed by a circular corrugated feed-horn (placed in the focal point of the dish) is considered here. An offset configuration is chosen in order to avoid the obstruction and scattering of the feed system [18] (p. 40).

A very important parameter in the sizing of the main reflector is the taper edge, *i.e.*, the power of the Gaussian beam outside a given radius. In [18] (p. 40), the offset reflector is designed considering the parent paraboloid. The same method will be followed here. The projection of the dish shape on the output plane is a circle of radius *r*. This value is useful for the computation of the output taper edge $t_{e}$, *i.e.*, as follows:

(5) $$t_{e}=\exp\left[-2\;{\left(\frac{r}{w}\right)}^{2}\right]$$

Since, with the parent paraboloid design approach, the output plane is also the beam waist location (planar phase front), the above equation can be computed for r=a and $w=w_{0}$. As a consequence, Equation (5) can be rewritten as:

(6) $$t_{e}=\exp\left[-2\;{\left(\frac{a}{w_{0}}\right)}^{2}\right]$$

As an example, for a taper edge of −27 dB and for a frequency of 31.4 GHz, one obtains a = 120.9 mm. This value can be used an an initial guess for a further dish optimization. Note that a taper edge equal to −27 dB means that only 1/500 of the total power in the Gaussian beam is not intercepted by the antenna reflector. This also guarantees a low side-lobes level. Table 3 reports some numerical examples. In this table, $l_{F}$ is the focal length of the dish and *D* is its diameter.

## 4. Antenna Noise Temperature

The antenna noise temperature $T_{A}$ is an important parameter affecting, together with the receiver noise temperature, the system resolution. Such a temperature depends on the scene observed by the antenna and can be evaluated according to [15] (p. 144) as:

(7) $$T_{A}=\frac{A_{e}}{\lambda^{2}}\;\int \;\;\;\int_{\Omega }\;P_{n}\left(\rho ,\varphi \right)\;T_{B}\left(\rho ,\varphi \right)\;d\Omega$$

*λ* being the signal wavelength, $A_{e}$ the effective area of the antenna, $P_{n}$ its normalized radiation diagram, $T_{B}$ the brightness temperature of the observed source and Ω the whole solid angle (*i.e.*, 4π Steradian). The variables *ρ* and *ϕ* are used to describe the spatial variations of the above functions.

In the case of fire detection, the brightness temperature $T_{B}$ can be written as:

(8) $$T_{B}\left(\rho ,\varphi \right)=\left\{\begin{array}{cc} T_{F} & \Omega_{F}\\ T_{S} & \Omega -\Omega_{F} \end{array}\right.$$

where $T_{F}$ and $T_{S}$ are the brightness temperatures of fire and soil (for soil, we mean the background), respectively. With such a scheme, the antenna is imagined at the center of a spherical surface. This surface is at $T_{S}$ except the fire spot $\Omega_{F}$, which is at $T_{F}$. The antenna beam is exactly pointed toward the fire spot. Using Equation (8), the above integral becomes:

(9) $$T_{A}=T_{F}\;\frac{A_{e}}{\lambda^{2}}\;\int \;\;\;\int_{\Omega_{F}}P_{n}\left(\rho ,\varphi \right)\;d\Omega \;+\;T_{S}\;\frac{A_{e}}{\lambda^{2}}\;\int \;\;\;\int_{\Omega -\Omega_{F}}\;\;\;\;\;\;P_{n}\left(\rho ,\varphi \right)\;d\Omega$$

Now, in the approximation of small fires, $\Omega -\Omega_{F}\approx \Omega$, thus:

(10) $$T_{A}\approx T_{F}\;\frac{A_{e}}{\lambda^{2}}\;\int \;\;\;\int_{\Omega_{F}}P_{n}\left(\rho ,\varphi \right)\;d\Omega \;+\;T_{S}\;\underset{1}{\underset{︸}{\frac{A_{e}}{\lambda^{2}}\;\int \;\;\;\int_{\Omega }P_{n}\left(\rho ,\varphi \right)\;d\Omega }}$$

and using the definition of normalized radiation pattern:

(11) $$T_{A}\approx T_{S}\;+\;T_{F}\;\frac{A_{e}}{\lambda^{2}}\;\int \;\;\;\int_{\Omega_{F}}P_{n}\left(\rho ,\varphi \right)\;d\Omega$$

**Figure 3:**
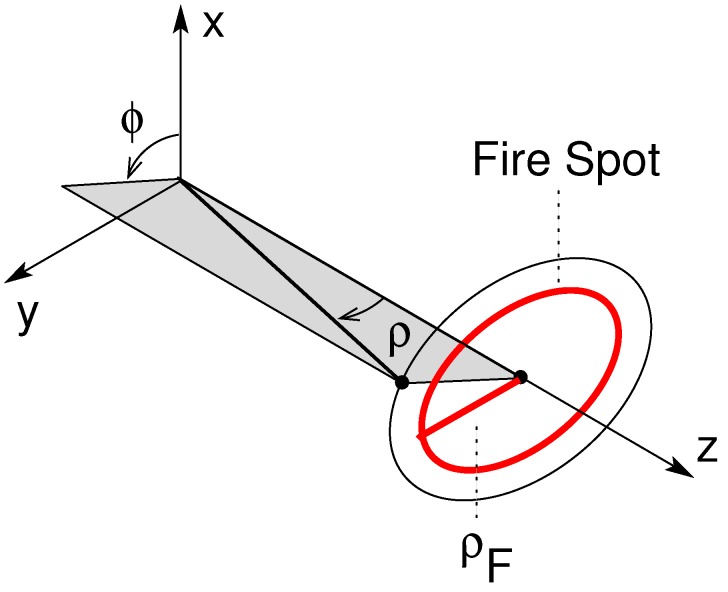
Coordinate system used to define the intensity distribution over the fire spot. A circular fire shape is assumed. The antenna is pointed toward the fire center.

For the computation of the remaining integral, $\Omega_{F}$ must be determined. For this purpose, it is particularly convenient to define *ρ* as the angular coordinate along the fire spot radius and *ϕ* as the angular coordinate around the antenna-to-fire axis (see Figure 3). In the case of fires with circular shape and angular radius $\rho_{F}$, the domain $\Omega_{F}$ is defined by the following inequalities. As a consequence,

(12) $$\Omega_{F}=\left\{0\leq \rho \leq \rho_{F},\;0\leq \varphi \leq 2\;\pi \right\}$$

The differential of the solid angle is computed as:

(13) dΩ=sinρdρdφ

Note that, in the case of small fires, *i.e.*, fires at the beginning of their activity, the small-angle approximation holds and the differential solid angle become:

(14) dΩ≈ρdρdφ

where $\rho \leq \rho_{F}\ll \Theta_{h}$ and $\Theta_{h}$ is the above introduced half-power beam-width of the antenna. Substituting Equation (14) in Equation (11), one gets:

(15) $$T_{A}\approx T_{S}\;+\;T_{F}\;\frac{A_{e}}{\lambda^{2}}\;\int_{0}^{2\;\pi }\;\;\;\int_{0}^{\rho_{F}}P_{n}\left(\rho ,\varphi \right)\;\rho \;d\rho \;d\phi \approx T_{S}\;+\;2\;\pi \;T_{F}\;\frac{A_{e}}{\lambda^{2}}\;\int_{0}^{\rho_{F}}\rho \;d\rho$$

The approximation is equivalent to consider equal to unity the normalized radiation pattern within $\Omega_{F}$. Developing the integral Equation (15), the following formula is derived:

(16) $$T_{A}\approx T_{S}\;+\;\pi \;\frac{A_{e}\;\rho_{F}^{2}}{\lambda^{2}}\;T_{F}$$

At this point, it is useful to recall that the antenna theorem states a basic equation between the effective antenna area and the antenna solid angle $\Omega_{a}$, [19] (p. 157):

(17) $$A_{e}\;\Omega_{a}=\lambda^{2}$$

Furthermore, for a Gaussian beam, it is possible to relate the antenna solid angle with the far-field divergence angle [15] (p. 136), and the latter with the half power beam width [15] (p. 25); one then obtains:

(18) $$\Omega_{a}=\frac{\pi }{2}\;{\left(\frac{\Theta_{h}}{2\;\sqrt{\ln\sqrt{2}}}\right)}^{2}=\frac{\pi }{2\;\ln\sqrt{2}}\;{\left(\frac{\Theta_{h}}{2}\right)}^{2}$$

Note that $2\;\sqrt{\ln\sqrt{2}}\approx 1.18$ as reported in [15] (p. 25). Inserting Equations (17) and (18) into Equation (16), one obtains the antenna noise temperature in terms of system parameters:

(19) $$T_{A}\approx T_{S}\;+\;0.69\;{\left(\frac{2\;\rho_{F}}{\Theta_{h}}\right)}^{2}\;T_{F}=T_{S}\;+\;q\;T_{F},$$

where *q* is the filling factor, *i.e.*, the portion of the antenna solid angle filled by the source itself.

One can also observe that 0.69 is the numerical approximation of $2\;\ln\sqrt{2}$. Equation (19) means that the antenna temperature can be obtained as the brightness temperature of the source multiplied by the filling factor *q*. A numerical example is reported in Table 4. In this example, a fire spot of radius $r_{F}=5\;\mathrm{cm}$ (*i.e.*, of 10cm diameter) is placed at a distance d=4m. The antenna footprint is circular; corrections for an angled observation (elliptical footprint) are reported in [4]. In the case of an angled view, the antenna noise temperature is lower than that given by Equation (19).

## 5. Instrument Resolution

The resolution ΔT of a millimeter-wave radiometer is defined as the standard deviation of the measured antenna noise temperature. As a consequence, the resolution represents, in a statistical sense, the minimum temperature variation that can be detected by the instrument. The radiometer readout will stay within a range of ±3ΔT from the measured temperature with a probability equal to 99.7%.

The instrument resolution will be studied in three steps. First, a basic analysis will be carried-out. Then, important performances of the square-law power detector will be studied. Finally, the detector behavior will be introduced in the resolution analysis, and the initial estimation will be refined.

### 5.1. Basic Analysis

A Total Power Radiometer (TPR) is shown in Figure 2. The resolution of such an instrument has been described in [20]. In the first approximation, this parameter can be estimated with:

(20) $$\Delta T=\left(T_{A}+T_{R}\right)\;\;\sqrt{\frac{1}{B\;\tau }+{\left(\frac{\Delta G}{G}\right)}^{2}}$$

The importance of this expression is that it establishes a link between the standard deviation of the measured data and the main instrument parameters, particularly the antenna noise temperature $T_{A}$, the receiver equivalent noise temperature $T_{R}$, the pre-detection bandwidth *B*, the integration time *τ* and the gain stability ΔG/G of the radiometer itself. $T_{R}$ can be related to the receiver noise figure $F_{R}$ by:

(21) $$T_{R}=\left(F_{R}-1\right)\;T_{0}$$

$T_{0}=290\;K$ being the standard IEEE temperature at which the noise figure is defined. The resolution of the proposed instrument has been analyzed with reference to the system parameters reported in Table 5. Three receiver noise figures have been considered in the range between 4 dB to 8 dB. These values include the majority of the millimeter-wave systems up to date; thus, they are widely feasible with the present technologies [21].

**Figure 4:**
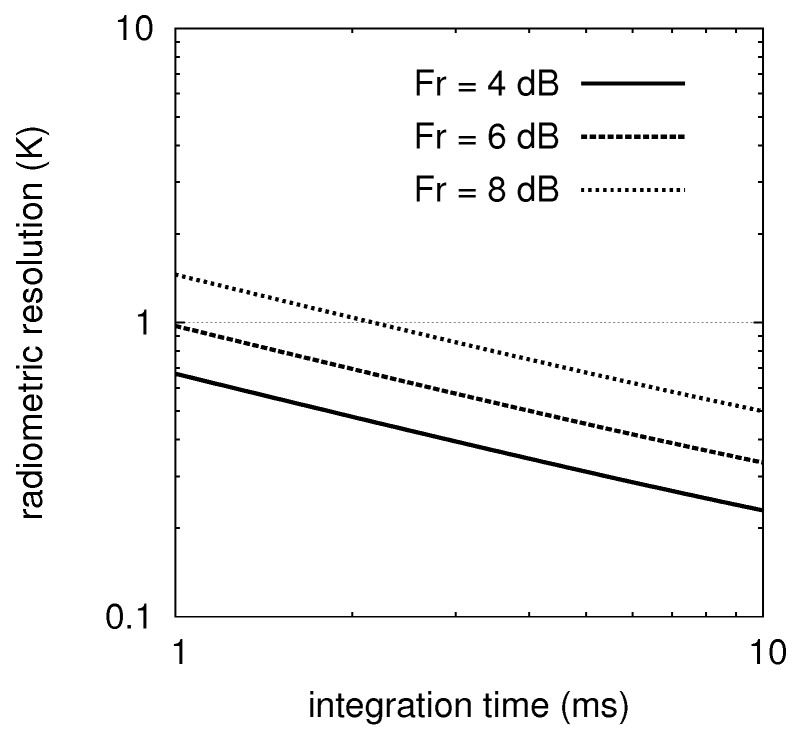
Radiometric resolution versus the integration time for $F_{R}$ from 4 dB to 8 dB. These noise figures are feasible at mm-waves with the present technology.

If the integration time is swept from 1 ms to 10 ms, the graphs of Figure 4 are obtained. Signal integration for more than 10 ms has not been considered because of the stringent requirements imposed by the train speed (see Table 1).

From the figure, it is clear that a 1.0 K resolution (for 1 ms integration time) can be achieved exploiting a receiver with a noise figure less than or equal to 6 dB, and featuring a 100 ppm overall gain stability. At this point, it is worth noticing that the 100 ppm value is 10 times larger than what is presently considered the state-of-the-art [22]. This effect (*i.e.*, low-frequency gain fluctuations of a microwave amplifier) has been experimentally characterized since 1996 and features a 1/f behavior [23]. To deal with the gain stability, the radiometer should be periodically calibrated. As a consequence, internal calibration standards will be provided, while a thermal stabilization of the instrument is generally needed.

### 5.2. Detector Performance

The square-law detector is a key building-block of a radiometric system. The purpose of this circuit is to generate an output voltage proportional to the power of the microwave signal injected at its input. The performance of the detector significantly affects that of the whole system, thus it will be briefly discussed here.

The principal detector figure-of-merit is the responsivity ${ℜ}_{d}$, which is given by (general case of a non-linear detector):

(22) $${ℜ}_{d}=\frac{\partial V_{d}}{\partial P_{d}}$$

where $V_{d}$ is the output (DC) voltage, $P_{d}$ is the power at the detector input and ${ℜ}_{d}$ is typically expressed in mV/μW. The responsivity is a very important parameter since it determines, together with the receiver gain, the sensitivity of the microwave radiometer.

When the power to be detected by the radiometer is very low, the noise generation mechanisms due to the real devices (diodes or transistors) used to implement the detector must be accounted for. This noise can be modeled as a voltage $v_{n}^{rms}$ additive with respect to the detected voltage $V_{d}$. Its Root Mean Square (RMS) voltage spectral density is well approximated by:

(23) $$S_{n}\left(f\right)=k_{f}\;\frac{V_{d}}{\sqrt{f}}\;+\;k_{w}$$

where $V_{d}$ accounts for the self-biasing of the detector. According to [24], it can be observed that the spectrum often follows the $1/f^{a}$ law, where *a* is close to, but slightly different from, unity. Here, a=1 is assumed for simplicity. In Equation (23), $S_{n}\left(f\right)$ is expressed in $V/\sqrt{\mathrm{Hz}}$, while $k_{f}$ and $k_{w}$ are the flicker (or 1/f) and white noise parameters, respectively.

**Figure 5:**
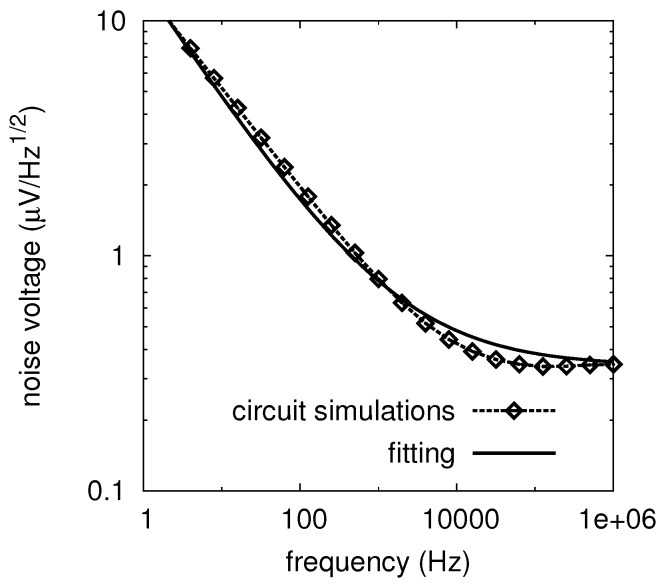
Low-frequency output noise voltage for a 36.8 GHz square-law detector, after [25]. The detector is realized using a differential configuration with two Hetero-junction Bipolar Transistors (HBTs). The detection is obtained using the non-linear characteristics of the HBT. The transistors are biased with a collector current of about 750μA. The input power is equal to 0.63μW, *i.e.*, −32 dBm. The corresponding output voltage is $V_{d}=60\;\mathrm{mV}$. The circuit simulations are compared with the standard Flicker noise model given in Equation (23) with the parameters of Table 6.

The typical behavior of $S_{n}\left(f\right)$ is shown in Figure 5, where a biased detector is considered. Such a circuit uses a differential configuration with two Hetero-junction Bipolar Transistors (HBT), and the detection is obtained exploiting their non-linear characteristics. Additional details about this specific design can be found in [25]. Using a fitting procedure, it is possible to determine the noise parameters according to Equation (23): these parameters are quoted in Table 6.

The data of Zero-Bias Schottky diodes in Table 6 have been taken from [26]. These diodes are produced by *Virginia Diodes* exploiting a GaAs process and constitute the state-of-the-art in millimeter-wave detection. In particular, the 1/f noise model reported in the table has been extracted from the measurements at 10kHz video frequency.

A very important detector figure of merit is the Noise-Equivalent Power (NEP). It is defined as the detector output RMS noise voltage spectral density $S_{n}\left(f\right)$ divided by the responsivity ${ℜ}_{d}$, e.g.,

(24) $${\mathrm{NEP}}_{d}\left(f\right)\;=\;\frac{S_{n}\left(f\right)}{\partial V_{d}/\partial P_{d}}\;=\;\frac{S_{n}\left(f\right)}{{ℜ}_{d}}$$

${\mathrm{NEP}}_{d}$ is expressed in $W/\sqrt{\mathrm{Hz}}$. Using the NEP, a detector can be treated as a noise-less component, and all its noise can be attributed to an equivalent input power (*i.e.*, with the same effects at the output).

### 5.3. Advanced Considerations

The detector noise is an important parameter determining the resolution of a radiometric system. If biased devices (diodes and transistors) are used, the Flicker noise component will dominate at low frequencies. As a consequence, switched instrument architectures, such as the Dicke radiometer [27], have to be considered. This is because of their inherent capability to reject the low frequency noise.

To start studying the impact of the detector noise on the instrument resolution, one has to consider the literature of the last ten years [28,29,30,31]. According to these papers, Equation (20) can be rewritten as follows:

(25) $$\Delta T=\beta \;\left(T_{A}+T_{R}\right)\;\;\sqrt{\frac{1}{B\;\tau }+{\left(\frac{\Delta G}{G}\right)}^{2}+{\left[\;\frac{N_{d}}{k_{B}\;\left(T_{A}+T_{R}\right)\;B\;G}\;\right]}^{2}}$$

where $k_{B}=1.38\times 10^{-23}$ J/K is the Boltzmann constant, *G* is the power gain of the receiver front-end (*i.e.*, the blocks in front of the detector), *β* is a constant equal to 2 for Dicke radiometers and equal to 1 for total-power radiometers and $N_{d}$ is the equivalent detector noise power referred to the detector input. $N_{d}$ is obtained with a frequency domain integration:

(26) $$N_{d}=\frac{1}{{ℜ}_{d}}\;\sqrt{\left[\;\int_{0}^{\;\infty }\;S_{n}^{2}\left(f\right)\;{\left|H(f)\right|}^{2}\;df\;\right]\;}$$

H(f) being the frequency response of the integrator and of all the signal processing carried-out at the radiometer output. In the previous integration, ${ℜ}_{d}$ is assumed to be constant over the video bandwidth and, thus, has been pulled out of the integral sign. It is useful to observe, however, that ${ℜ}_{d}$ is dependent on the observation frequency, *i.e.*, the microwave frequency at which the radiometer operates. According to Equation (26), $N_{d}$ represents the equivalent power at the detector input that generates, at the detector output, a DC voltage equal to the detector RMS noise voltage.

Coming now back to Equation (25), one can observe that $N_{d}/\left(k\;B\;G\right)$ is the detector noise power referred to the radiometer input ($N_{d}$ is divided by the front-end power gain *G*) and expressed in terms of equivalent noise temperature using the Johnson–Nyquist model (the Boltzmann constant *k* and the equivalent noise bandwidth *B* of the radiometer are at the denominator as well). We can thus imagine that:

(27) $$\Delta T_{d}=\frac{N_{d}}{k_{B}\;B\;G}$$

is the equivalent temperature fluctuation that needs to be accounted for, at the input, to model the detector noise. Since this fluctuation is statistically independent from the other noise sources, it is squared summed in Equation (25) to get the final result. The formulation shown above is mathematically equivalent to that found in [32].

### 5.4. Dicke Radiometers with Detector Noise

In the first case, a Dicke radiometer (DK) is assumed (see again the schematic in Figure 2). For this purpose, the radiometer input is periodically switched between the antenna at temperature $T_{A}$ and a black-body at temperature $T_{BB}$. At the same time, the detector output is multiplied by ±1 in synchronism with the receiver input switching (*i.e.*, with an approach similar to that used by lock-in amplifiers). If the switching frequency $f_{m}$ is sufficiently high and $T_{BB}≃T_{A}$ (same order of magnitude), the gain fluctuations ΔG/G can be neglected in Equation (25) and the resolution approximated by:

(28) $$\Delta T=2\;\left(T_{A}+T_{R}\right)\;\;\sqrt{\frac{1}{B\;\tau }+{\left[\;\frac{N_{d}}{k_{B}\;\left(T_{A}+T_{R}\right)\;B\;G}\;\right]}^{2}}$$

The main advantage of the Dicke configuration is that the 1/f noise spectrum at the detector output is modulated around $\pm f_{m}$ (and its harmonics). As a consequence, the noise contribution within the integrator bandwidth (equal to 1/2τ) is significantly reduced. The main disadvantage, instead, is that the scene is observed for only half of the operating time. Thus, the resolution of a Dicke radiometer is only half that of an ideal radiometer (with the same *B* and *τ* parameters). This is accounted for in Equation (28) assuming β=2 and results, equivalently, in the ΔT doubling.

To derive the equivalent detector noise power $N_{d}$ to be substituted in Equation (28), one can imagine that the Dicke modulation and the subsequent integration is equivalent to the following frequency response:

(29) $${\left|H(f)\right|}^{2}\approx \left\{\begin{array}{cc} 1 & f_{L}<f<f_{H}\\ 0 & \mathrm{elsewhere} \end{array}\right.$$

where $f_{L}\approx f_{m}-1/4\tau$ and $f_{H}\approx f_{m}+1/4\tau$. Note that 1/2τ is the bandwidth of the ideal integrator. From Equation (26), we have:

(30) $$N_{d}=\frac{1}{{ℜ}_{d}}\;\sqrt{\left[\;\int_{f_{L}}^{\;f_{H}}\;\;S_{n}^{2}\left(f\right)\;df\;\right]\;}\approx \frac{1}{{ℜ}_{d}}\;\sqrt{\left[\;\frac{1}{2\;\tau }\;S_{n}^{2}\left(f_{m}\right)\;\right]\;}=\frac{S_{n}\left(f_{m}\right)}{\sqrt{2\;\tau }\;{ℜ}_{d}}$$

The results have been obtained considering $S_{n}\left(f\right)$ constant in the integration interval. Furthermore, $f_{m}$ is generally assumed much greater than the corner frequency of the 1/f noise. As a consequence, both of the Low-Noise Amplifiers (LNAs) gain fluctuations (*i.e.*, ΔG/G), and the Flicker noise of the detector can be neglected. Exploiting Equation (24), one gets:

(31) $$N_{d}=\frac{{\mathrm{NEP}}_{d}\left(f_{m}\right)}{\sqrt{2\;\tau }}$$

### 5.5. Total Power Radiometers with Detector Noise

In the second case, a total power radiometer (TPR) is considered. In order to determine the resolution, however, an equivalent parameter must be used in the place of $NEP_{d}$. The starting point is a fundamental work by Hersman and Poe [20], where the low-frequency behavior of a TPR instrument is studied in depth. In particular, the TPR is modeled along with the calibration process. This process consists of periodically switching the receiver input on two loads at different temperatures, namely $T_{1}$ and $T_{2}$ with $T_{1}<T_{A}<T_{2}$. In this way, the receiver gain constant (expressed as $kBG{ℜ}_{d}$) and the receiver equivalent noise temperature ($T_{R}$), which are both unknown form an experimental point of view, can be determined. These instrument parameters will then be used, in the time interval between two calibrations, to relate the output detector voltage $V_{d}$ to the input antenna noise temperature $T_{A}$.

One of the main conclusions reported in [20] is that the combined action of such a calibration process and of the radiometer integrator is equivalent to a low-frequency band-pass filter. The frequency response of this filter depends on both the calibration period $t_{c}$ and the integration time *τ* and can approximated by:

(32) $${\left|H(f)\right|}^{2}\approx \left\{\begin{array}{cc} 2 & f_{L}<f<f_{H}\\ 0 & \mathrm{elsewhere} \end{array}\right.$$

where $f_{L}\approx 1/2t_{c}$, $f_{H}\approx 1/2\tau$ and $\tau \ll t_{c}$. In particular, Equation (32) neglects the frequency roll-off below $f_{L}$ and above $f_{H}$, since these follow the $1/f^{2}$ law. Furthermore, the same integration time *τ* is assumed to be used during the calibration and measurement phases.

Following the method outlined in [20], it is easy to conclude that the noise voltage at the output of the filter H(f) is:

(33) $$\bar{\Delta v_{n}^{2}}=\int_{\;0}^{\;\infty }\;S_{n}^{2}\left(f\right)\;{\left|H(f)\right|}^{2}\;df$$

Now, substituting Equations (32) and (23) in Equation (33), one can evaluate the above integral as a function of the system parameters:

(34) $$\bar{\Delta v_{n}^{2}}\approx 2\;\int_{\;f_{L}}^{\;f_{H}}\;\left(\frac{k_{f}^{2}\;V_{d}^{2}}{f}+k_{w}^{2}\right)\;df=2\;k_{w}^{2}\;\left(f_{H}-f_{L}\right)+2\;k_{f}^{2}\;V_{d}^{2}\;\ln\left(\frac{f_{H}}{f_{L}}\right)$$

Observing that $f_{H}-f_{L}\approx f_{H}$, $f_{L}\approx 1/2t_{c}$, $f_{H}\approx 1/2\tau$ and using the triangular inequality:

(35) $$v_{n}^{tpr}=\sqrt{\;\bar{\Delta v_{n}^{2}}\;}\leq \frac{k_{w}}{\sqrt{\tau }}+k_{f}\;V_{d}\;\sqrt{2\;\ln\left(\frac{t_{c}}{\tau }\right)}$$

where $v_{n}^{tpr}$ is an integral voltage obtained by considering the band-pass filter associated to the TPR data processing. The filter bandwidth is limited by both the calibration periodicity $t_{c}$ (low cut-off frequency) and integration time *τ* (high cut-off frequency). In order to use the above results in Equation (25), however, the responsivity ${ℜ}_{d}$ must be considered. Exploiting Equation (26), one gets:

(36) $$N_{d}=\frac{v_{n}^{tpr}}{{ℜ}_{d}}\;\leq \;\frac{1}{\sqrt{2\;\tau }\;{ℜ}_{d}}\left[\sqrt{2}\;k_{w}+2\;k_{f}\;V_{d}\;\sqrt{\tau \;\ln\left(\;\frac{t_{c}}{\tau }\;\right)}\;\right]$$

## 6. Preliminary System Design

The resolution analysis shown in Figure 4 is based on two main assumptions: the receiver noise figure is less than 6dB and gain fluctuations are below 100ppm. In order to complete the radiometer system design, the following steps have been carried-out. First, the overall receiver gain is evaluated. Then, gain and noise figures are distributed along the chain of Figure 2. The gain is determined by selecting a suitable power level $P_{d}$ at the square-law detector input:

(37) $$P_{d}=k_{B}\;\left(T_{A}+T_{R}\right)\;B\;G$$

where, as above, *B* and *G* are the pre-detection noise bandwidth and gain, respectively. Combining Equation (37) with Equation (22), the antenna noise temperature can be related with the output radiometer voltage:

(38) $$V_{d}=k_{B}\;B\;G\;{ℜ}_{d}\;\left(T_{A}+T_{R}\right)$$

Note that the above result is obtained assuming a linear detector, *i.e.*, a detector for which the responsivity is independent of the input power. Equation (38) is an important link between the quantity to be measured, $T_{A}$, and the quantity effectively handled, $V_{d}$. In particular, $k\;B\;G\;{ℜ}_{d}$ plays the role of a temperature-to-voltage conversion constant, $T_{A}$ is the wanted signal, while $T_{R}$ constitutes the radiometric offset. The derivative of $V_{d}$ with respect to $T_{A}$ defines the radiometer sensitivity, *i.e.*, the variation of the output voltage associated to a 1K variation of the antenna noise temperature:

(39) $$\frac{\partial V_{d}}{\partial T_{A}}=k_{B}\;B\;G\;{ℜ}_{d}$$

The minimum variation of the output voltage $\Delta V_{d}^{min}$ is obtained multiplying the radiometer sensitivity Equation (39) by the radiometer resolution Equation (20). Such a value should be kept well above the input offset and the thermal drift stability of the video amplifier, *i.e.*, of the operational amplifier following the square-law detector.

To meet such a criterion, it is useful to improve the radiometer sensitivity, which, in turn, can be done improving both the detector responsitivity and the pre-detection gain. The detector sensitivity is related to the semiconductor material used in the detector diode. Typical values of ${ℜ}_{d}$ for broadband detectors are 0.5mV/μW for Si Schottky diodes and 1mV/μW for GaAs Schottky diodes. Tuned zero-bias Schottky [26] or tunnel [29] diodes can achieve a responsivity as high as 3–10mV/μW. Transistor-based detectors in CMOS or SiGe BiCMOS technologies, on the other hand, are capable of higher responsivities at the expense of large noise levels [33,34]. For this reason, the two last technologies will not be considered, for the moment, in the system analysis.

The pre-detection gain should be determined in such a way as to guarantee the linearity of the detector. For this purpose, $P_{d}$ should be kept below −27 dBm for Si diodes and below −18 dBm for GaAs diodes. Using these data and considering the specifications defined in Table 1, the values quoted in Table 7 are obtained. In particular, the noise power at the radiometer input $k\;(T_{A}+T_{R})\;B$ is −74.2 dBm (equal to 37.7μW for $T_{A}=500\;K$ and $T_{R}=865\;K$), and the radiometric resolution is 1.0 K.

From the analysis of Table 7, the selection of a GaAs detector diode along with an overall pre-detection gain of about 56dB comes out. The offset and drift parameters of the Instrumentation Amplifier (IA) have been taken from the AD524C data-sheet [35] and have been reported hede for comparison with the minimum detected voltage $\Delta V_{d}^{min}$.

To distribute gain and noise figures along the radiometer chain, the performance of off-the-shelf mm-wave components have been evaluated. A summary of this study is illustrated in Table 8. In particular, a single HMC566 integrated amplifier from Hittite [36] has been considered as the first LNA stage. This circuit features a gain of about 22dB and a noise figure of 2.8dB (typical mean values taken from the data-sheet) at 32GHz. After the band-pass filter, three more stages are used to boost the receiver gain at the desired level. In this case, the HMC519 is selected [37]. This device has a gain of 14 dB per stage (typical) with a noise figure of 4.5dB (worst case). Three stages in cascade thus feature an overall 42dB gain with a 4.6 dB noise figure. An integrated Single Pole Double Trow (SPDT) switch, such as the HMC971 device [38], can be used as Dicke switch. Such a device features an insertion loss of about 1.3 dB.

The noise figure of the whole receiver $F_{R}$ can be evaluated, according to the Friis formula, as a function of the single blocks performance. Developing this well-known relationship, one obtains:

(40) $$F_{R}=L_{WG}\;L_{SW}\;L_{CP}\;L_{IS}\;F_{LNA}^{\left(1\right)}+\frac{L_{WG}\;L_{SW}\;L_{CP}\;L_{IS}}{G_{LNA}^{\left(1\right)}}\;\left(L_{BPF}\;F_{LNA}^{\left(2\right)}-1\right)$$

where $L_{j}=1/G_{j}$ is the loss of the generic passive block. The above expression uses the following notation: WG = waveguide adapter; SW = Dicke switch; CP = noise injection directional coupler; IS = isolator; LNA = Low Noise Amplifier; BPF = Band-Pass Filter. For the passive blocks, the noise figure has been assumed equal to the insertion loss.

Exploiting the numerical values of the above table and converting the dB quantities in linear units, one obtains:

(41) $$F_{R}=3.80+\frac{1.58}{100}\;\left(7.08-1\right)=3.90\;\;\;\;⟶\;\;\;\;5.9\;\mathrm{dB}$$

As a result, it can be concluded that, with the selection of commercial component proposed in Table 8, the $F_{R}<6\;\mathrm{dB}$ performance can be achieved at the considered operating frequency. A better noise figure can be obtained exploiting the modified Dicke architecture suggested in [39].

## 7. Calibration and Processing

The calibration is a fundamental procedure allowing the antenna noise temperature $T_{A}$, *i.e.*, the quantity under measurement, to be evaluated in terms of the output radiometer voltage, $V_{m}$, that is directly measured by the Analog to Digital Converter (ADC) connected to the micro-controller. To cope with this issue, a calibration circuitry mathematical model was developed. The interested readers can refer to [40] (pp. 357–384) for a complete description of this issue. The structure of the software needed to manage the radiometer and to acquire the brightness temperature data, instead, is found in [4] (pp. 2636).

## 8. Implementation Hypothesis

In this section, two implementation hypotheses will be analyzed and discussed more in depth. The feasibility analysis will be extended to the availability of both commercial components and viable technologies. In particular, the following possibilities will be considered:

- a 30 GHz radiometer implemented by means of GaAs “off-the-shelf” Integrated Circuits (ICs);
- a 24 GHz radiometer based on discrete components and single Printed Circuit Board (PCB) solution.

It is worth noticing that the last instrument was developed at the University of Perugia, thanks to two Ph.D. theses [41,42].

### 8.1. GaAs Off-the-Shelf ICs

The first implementation hypothesis is the one already proposed in Table 8. It uses off-the-shelf GaAs integrated circuits to implement all the critical building blocks of the mm-wave receiver. In addition, GaAs Zero-Bias Schottky diodes will be adopted for the power detector, whereas noise diodes (exploiting the excess noise generated when a junction is driven in breakdown) will be used as noise sources for the receiver gain calibration. Finally, high performance operational amplifiers and CPU boards with on-board ADC (typically with Linux embedded operating system) are available to complete the radiometer. In summary, all of the critical components listed below can be directly acquired from the market, thus demonstrating the feasibility of the radiometer.

- HMC971 (Hittite) for the Dicke switch;
- HMC566 (Hittite) for the first LNA stage;
- HMC519 (Hittite) for the remaining LNA stages;
- W-Band ZBD diodes (Virginia Diodes) for the power detector;
- noise diodes (Noisecom) for the gain calibration;
- standard rectangular waveguide (WR28) components;
- AD524 (Analog Device) for the operational amplifier;
- Red Pitaya for the CPu board with Linux embedded OS.

For the antenna, commercial solutions might exist because the 28GHz band is used for radio-links. If this is not the case, a custom design must be developed. Similarly, some WR28 waveguide component, the PCBs and the mm-wave receiver packaging must be designed for the specific application.

### 8.2. Discrete-Component Technology

**Figure 6:**
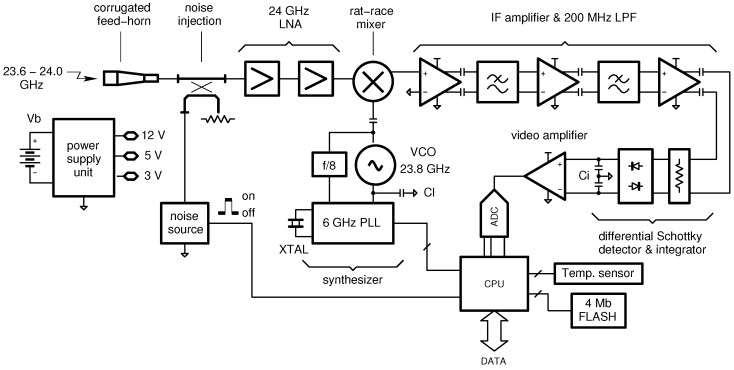
Architecture of the developed single-board, 24-GHz, Noise Adding Radiometer (NAR). For low-cost applications, the calibration circuitry of Figure 2 is simplified by removing the Dicke switch. This means that only the gain is corrected. The radiometric offset is less important since, in fire detection, the information is carried by the radiometric contrast (brightness temperature difference).

Figure 6 shows the block diagram of a 24GHz radiometer, recently developed at the University of Perugia [41,42]. Such an instrument is based on the Noise Adding Radiometer (NAR) architecture and exploits a homo-dyne receiver along with a discrete-component technology.

A target noise figure of 2.5 dB (∼220 K) is achievable currently with Ku-band LNA. Inverting the radiometer resolution equation and using an integration time of 10ms, a theoretical resolution ΔT=0.2K is possible. An overall gain of about 70dB is necessary to meet the optimum detector input power range with about 100MHz of Intermediate Frequency (IF) bandwidth. The LNA gain is around 20dB. The 24.0GHz front-end consists of a double stage LNA followed by a single-balanced mixer. A tunable synthesizer (23.0–24.5 GHz) has been realized exploiting an integrated VCO [43]. This device also includes a three stage buffer amplifier and a divider by eight. The frequency stabilization is achieved with a fractional-N PLL [44] that locks the VCO with a 26MHz Temperature Compensated Crystal Oscillator (TCXO) capable of 5ppm frequency stability [45].

A microprocessor unit, based on a 8051 CPU [46], is used to sample the detected video signal by means of the on-chip 24bit ADC. The CPU is thus programmed to acquire data up to 1Ksps speed and to implement the signal integration (*i.e.*, averaging) directly in the digital domain. With the number of samples per measure is fixed at 100, the integration time can be controlled at firmware-level by simply adjusting the sampling frequency of the ADC. In the present version, the integration time can be varied between 0.1s and 10s. In parallel with measurement operation, the CPU is also used for other crucial tasks such as calibration, data storage on a flash memory and serial communication with the host PC.

**Figure 7:**
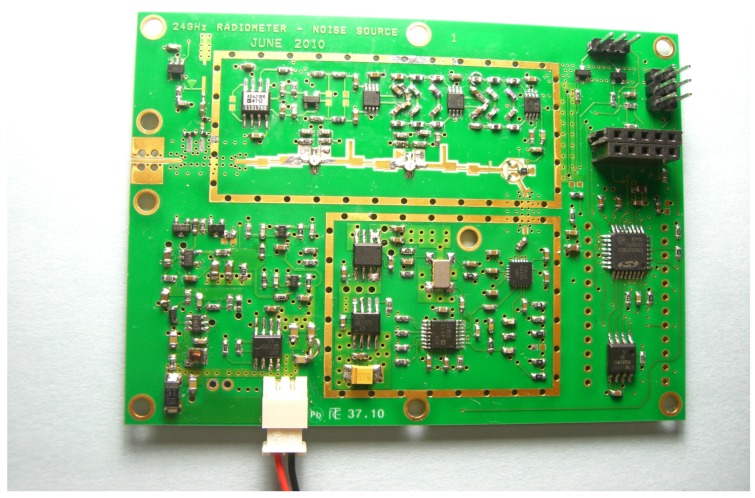
Photo of the single-board, 24-GHz radiometer prototype.

A noise source is realized with a noise diode [47] inverse biased to the breakdown region; it is capable of generating noise up to 26GHz. Noise injection is performed by a strip line directional coupler realized directly on to the PCB, exploiting the four-layer stack-up. From a technological point of view, the instrument is implemented on the single PCB illustrated in Figure 7. The circuit receives the supply from a Lithium Polymer (LiPo) battery and has an autonomy of about 6 h.

As a final observation, it is interesting to underline that fire detection by microwave radiometers has already been demonstrated experimentally by several authors. The 12 GHz observations reported in [4], for example, show a radiometric contrast up to about 9 K for a wooden fire at 30 m from the antenna. The estimated ember temperature in this case was about 1100 K with a filling factor *q* less than 1%. Concerning the fire detection in the presence of obstacles/walls, an outdoor experiment with the same 12 GHz radiometer was carried out in [6] with a 2-cm thick plywood wall. Figures 6 and 9 in [6] show a radiometric contrast of 5 K caused by a small fire ($0.1\;m^{2}$ area) with an unfavorable filling factor *q* less than 5%.

## 9. Conclusions

In this work, for the first time, the theory of microwave radiometers for fire detection in moving trains and vehicles has been developed. The instrument design should account for system parameters such as the observation frequency, angle and distance, the vehicle speed, the window size, the antenna beam-width and the temperature resolution. As a case of study, a radiometer operating at 31.4 GHz is considered. Such an instrument uses a multi-beam configuration with three-pixels and a 34 cm offset parabolic dish. Each pixel is equipped with a direct amplification microwave receiver featuring a 2 GHz observation bandwidth, a 1 ms integration time and a 6 dB noise figure. Such a receiver is calibrated by means of both a Dicke switch (for the offset) and by noise injection (for the gain) and can achieve a 1 K brightness temperature resolution. Finally, two implementation hypotheses have been investigated showing that off-the-shelf integrated circuits and/or discrete components can be adopted for the implementation of the above radiometer. In particular, the 31.4 GHz receiver should rely on integrated circuits, whereas, if discrete components are used, the frequency cannot exceed 26 GHz, since most of the packaged active components (*i.e.*, transistors and diodes), are not available above this threshold.

## Figures and Tables

**Table 1:** Specifications.

$f_{0}$ (GHz)	*B* (GHz)	$N_{b}$	$\Theta_{h}$ (deg.)	*d* (m)	$v_{T}$ (km/h)	*τ* (ms)	ΔT (K)	$T_{A}$ (K)
31.4	2	3–5	3	2–4	up to 300	1–10	1.0	0–500

**Table 2:** Gaussian optics parameters.

$\Theta_{h}$ (deg.)	$\theta_{0}$ (deg.)	$f_{0}$ (GHz)	*λ* (mm)	$w_{0}$ (mm)	$z_{c}$ (m)
3	2.54	31.4	9.55	68.6	1.55
3	2.54	24.0	12.5	89.8	2.03
3	2.54	12.0	25.0	179.5	4.05
3	2.54	10.0	30.0	215.4	4.86

**Table 3:** Main reflector parameters.

$t_{e}$ (dB)	$f_{0}$ (GHz)	$w_{0}$ (mm)	*a* (mm)	$l_{F}/D$	$D=2\;\sqrt{2}\;a$ (mm)
−27	31.4	68.6	120.9	0.6	340
−27	24.0	89.8	158.3	0.6	448
−27	12.0	179.5	316.5	0.6	896
−27	10.0	215.4	379.8	0.6	1074

**Table 4:** Antenna noise temperature.

$T_{F}$ (K)	$T_{S}$ (K)	$r_{F}$ (cm)	*d* (m)	$\rho_{F}$ (deg.)	$\Theta_{h}$ (deg.)	$T_{A}$ (K)
1500	200	5	4	0.7	3	425

**Table 5:** Main instrument parameters.

$f_{0}$ (GHz)	$T_{A}$ (K)	$F_{R}$ (dB)	$T_{R}$ (K)	*B* (GHz)	*τ* (ms)	$\frac{\Delta G}{G}$ (ppm)	ΔT (K)
31.4	500	4	438	2	1	100	0.7
31.4	500	6	865	2	1	100	1.0
31.4	500	8	1540	2	1	100	1.5

**Table 6:** Typical detector noise parameters.

Technology	Reference	$f_{0}$ (GHz)	${ℜ}_{d}$ (mV/μW)	$k_{f}$	$k_{w}$ ($V/\sqrt{\mathrm{Hz}}$)	Notes
SiGe HBT	[25]	36.8	96	$2.3\times 10^{-4}$	$3.4\times 10^{-7}$	$P_{d}=0.63\;\mu W$
Zero-Bias Schottky	[26]	140	3.5	$6.0\times 10^{-5}$	$5.4\times 10^{-9}$	$P_{d}=15.8\;\mu W$

**Table 7:** Radiometer gain and sensitivity.

Type	$P_{d}$ (dBm)	${ℜ}_{d}$ (mV/*μ*W)	*G* (dB)	$\frac{\partial V_{d}}{\partial T_{A}}$ (*μ*V/K)	$\Delta V_{d}^{\min}$ (*μ*V)	IA Offset (*μ*V)	IA Drift (*μ*V/${}^{\circ }$C)
Si	−27	0.5	47.2	0.72	0.72	50	0.5
GaAs	−18	1.0	56.2	11.5	11.5	50	0.5

**Table 8:** 31.4 GHz radiometer building blocks breakdown.

Parameter	Unit	WG Adapter	Dicke Switch	Directional Coupler	Isolator	LNA Stage 1	BPF	LNA Stages 2–4
*G*	(dB)	−0.3	−1.3	−0.7	−0.7	22	−5.0	42
*F*	(dB)	0.3	1.3	0.7	0.7	2.8	5.0	3.5
$T_{eq}$	(K)	21	101	51	51	374	627	359
		WR-28	HMC971	WR-28	WR-28	HMC566	PCB	HMC519
